# *Myxovirus resistance* (*Mx*) Gene Diversity in Avian Influenza Virus Infections

**DOI:** 10.3390/biomedicines10112717

**Published:** 2022-10-27

**Authors:** Jahangir Alam, Md. Mostafizer Rahman, Joyanta Halder, Md. Rezuanul Islam, Nandini Sarkar, Ishrat Jabeen, Mridha Md. Kamal Hossain, Rubaya Rubaya, Md. Abdul Alim, Anjuman Ara Bhuyan, Nusrat Jahan, Md. Masudur Rahman, Hossam M. Ashour

**Affiliations:** 1 Animal Biotechnology Division, National Institute of Biotechnology, Dhaka 1349, Bangladesh; 2 Department of Microbiology, Gono Bishwabidyalay, Dhaka 1344, Bangladesh; 3 Department of Microbiology, Hajee Mohammad Danesh Science and Technology University, Dinajpur 5200, Bangladesh; 4 Department of Biotechnology and Genetic Engineering, Islamic University, Kushtia 7003, Bangladesh; 5 Department of Biochemistry and Microbiology, North South University, Dhaka 1229, Bangladesh; 6 Department of Pathology, Sylhet Agricultural University, Sylhet 3100, Bangladesh; 7 ABEx Bio-Research Center, East Azampur, Dhaka 1230, Bangladesh; 8 Department of Integrative Biology, College of Arts and Sciences, University of South Florida, St. Petersburg, FL 33701, USA

**Keywords:** *Myxovirus* resistance, *Mx* gene diversity, avian influenza, anti-AIV, AIV infections

## Abstract

Avian influenza viruses (AIVs) pose threats to animal and human health. Outbreaks from the highly pathogenic avian influenza virus (HPAIV) in indigenous chickens in Bangladesh are infrequent. This could be attributed to the *Myxovirus* resistance (*Mx*) gene. To determine the impact of *Mx* gene diversity on AIV infections in chicken, we assessed the *Mx* genes, AIVs, and anti-AIV antibodies. DNA from blood cells, serum, and cloacal swab samples was isolated from non-vaccinated indigenous chickens and vaccinated commercial chickens. Possible relationships were assessed using the general linear model (GLM) procedure. Three genotypes of the *Mx* gene were detected (the resistant AA type, the sensitive GG type, and the heterozygous AG type). The AA genotype (0.48) was more prevalent than the GG (0.19) and the AG (0.33) genotypes. The AA genotype was more prevalent in indigenous than in commercial chickens. A total of 17 hemagglutinating viruses were isolated from the 512 swab samples. AIVs were detected in two samples (2/512; 0.39%) and subtyped as H1N1, whereas Newcastle disease virus (NDV) was detected in the remaining samples. The viral infections did not lead to apparent symptoms. Anti-AIV antibodies were detected in 44.92% of the samples with levels ranging from 27.37% to 67.65% in indigenous chickens and from 26% to 87.5% in commercial chickens. The anti-AIV antibody was detected in 40.16%, 65.98%, and 39.77% of chickens with resistant, sensitive, and heterozygous genotypes, respectively. The genotypes showed significant association (*p* < 0.001) with the anti-AIV antibodies. The low AIV isolation rates and high antibody prevalence rates could indicate seroconversion resulting from exposure to the virus as it circulates. Results indicate that the resistant genotype of the *Mx* gene might not offer anti-AIV protection for chickens.

## 1. Introduction

Bangladesh is an agricultural country and livestock is an integral component of its agricultural economy. Livestock, including cattle, buffalo, sheep, goat and poultry, are a source of nutrition and manure and they provide a means of transport and income for many families. HPAI H5N1 infections have led to lower growth rates in poultry [1]. Indigenous chickens (~298.4 million) and ducks (~60.01 million) are important species of poultry that are mainly reared by women and provide a source of protein for families [2,3,4]. Livestock productivity in Bangladesh is negatively impacted by the absence of appropriate breeds for different livestock species including poultry, shortage of quality feeds and fodder, and inadequate veterinary coverage and technologies for disease diagnosis, treatment, and control [5].

An outbreak of HPAI occurred in Bangladesh in March 2007 causing the influenza virus to spread to different parts of the country [6,7,8,9] and to become endemic [10]. Continuous culling of infected chickens (estimated to be more than 250 million) caused an increase in food insecurity and slowed the economic growth [11,12,13]. Interestingly, a limited number of avian influenza outbreaks have been reported in indigenous chickens since the outbreak in 2007. Therefore, it is hypothesized that there might be some host factors that contributed to the lower number of HPAI outbreaks in indigenous chickens and ducks in Bangladesh. *Myxovirus* resistance gene (*Mx*) is one of the suspected host factors. The *Mx* gene coding for Mx protein confers resistance to *Orthomyxovirus* infection and has been found in yeast and vertebrates ranging from fish to humans [14]. Human *Mx*A has antiviral activity against *Orthomyxoviruses* including influenza viruses and *Paramyxoviridae* [15]. The mouse Mx1 protein, localized in the nucleus of the infected cell, was reported to primarily inhibit replication of Orthomyxoviruses including influenza viruses [16,17]. The chicken Mx protein, having serine at position 631 and also localized in the nucleus of the infected cell exhibits antiviral activity against influenza virus and recombinant vesicular stomatitis virus (VSV) [18]. Moreover, avian influenza resistance in indigenous Indonesian chickens through the *Mx* gene has been reported [19] 19. Interestingly, an asparagine 631 variant was found to be void of activity against H5N1 [20]. A single-point serine-to-asparagine mutation (AGC to ACC) in the Mx protein at position 631 has been reported to impact antiviral activity [21]. Other studies reported that the Mx gene was dispensable for antiviral activities [22,23,24,25]. Chickens in Bangladesh have at least two types of *Mx* gene alleles (A and G) and three genotypes (homozygous resistant, homozygous sensitive, and heterozygous) [26,27]. As the *Mx* gene may contribute to the lower occurrence of HPAIV in indigenous chickens, this study was conducted to determine the *Mx* gene diversity and its possible impacts on AIV infection in chicken.

## 2. Materials and Methods

### 2.1. Study Area and Chickens Investigated

**Figure 1:**
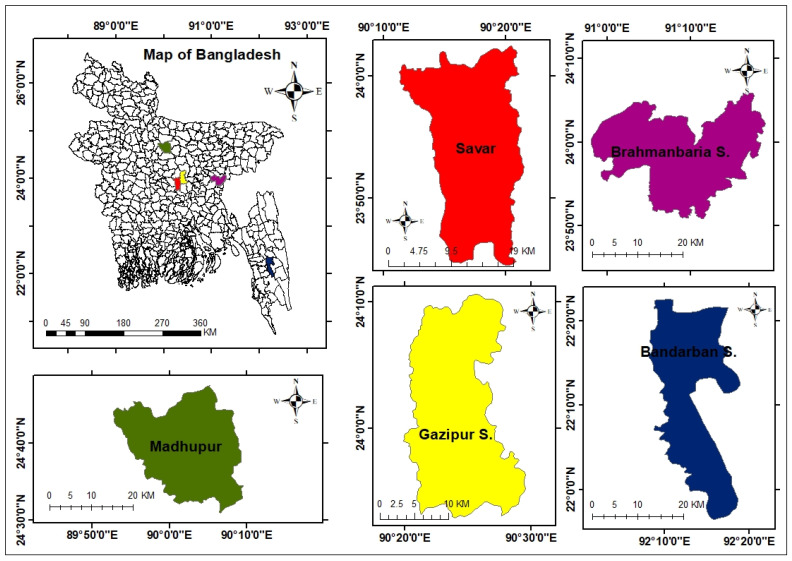
Location of the sampling sub-districts (upazila) of Bangladesh, marked by different colors. Map was created using ArcGIS 10.8.2. ArcGIS Enterprise, ESRI, Redlands, California, USA.

There are different types of indigenous chickens available in Bangladesh, such as Aseel, Hilly, Naked neck, Jungle fowl, Dwarf, and Non-descript deshi (Common deshi), Tiger birds, Creeper, Freezled, and Wrytail [3,28,29]. In the present cross-sectional study, Savar upazila of Dhaka district, Sadar upazila of Brahmanbaria district, Madhupur upazila of Tangail district, and Sadar upazila of Bandarban district were selected for sampling of Common deshi, Aseel, Naked neck, and Hilly chickens, respectively. Moreover, samples of commercial layer chickens were collected from Savar upazila of Dhaka district and Sadar upazila of Gazipur district (Figure 1). The study was approved by the Ethical Review Committee of the National Institute of Biotechnology, Dhaka 1349, Bangladesh. The handling of animals in the study was performed in accordance with the Cruelty to Animals Act 1920, Act No. I of 1920 of the Government of the People’s Republic of Bangladesh. 

### 2.2. Sample Collection

A total of 512 blood samples and 512 cloacal swab samples were collected from different types of indigenous and commercial chickens (Table 1). Blood and cloacal swab samples were collected from each chicken. After collection, the cloacal swab sample was kept in 1.5 mL of viral transport medium. The blood sample was kept at room temperature for the separation of serum and cellular portions. All samples were transported to the Animal Biotechnology Division (ABD), National Institute of Biotechnology (NIB) maintaining proper cold chain. From each blood sample, serum and cellular portions were separated into two samples. Thus, three types of samples were obtained, processed, and investigated for each chicken (blood cells, blood sera, and cloacal swabs) resulting in a total of 1536 samples. DNA was extracted from blood cells and assessed for the *Mx* gene and its diversity. Serum samples were tested for the anti-AIV antibody. Cloacal swab samples were tested for hemagglutinating virus isolation followed by the identification of AIV and Newcastle disease virus (NDV) by molecular testing.

### 2.3. DNA Extraction from the Cellular Portion of the Blood 

The cellular portion of the blood was used for *Mx* gene diversity analysis and genotyping of chickens. DNA was extracted via the phenol–chloroform method [30]. Briefly, 150 µL blood cells was retrieved from −20 °C sample, kept at room temperature until thawing and washed with 700 µL of de-ionized distilled water by mixing and centrifugation at 10,000 rpm for 10 min. The supernatant was discarded, and the process was repeated once. Then, 200 µL lysis buffer (0.5% SDS, 50 mM Tris–HCl pH 8 and 0.1 M EDTA) and 2 µL of Proteinase K were added and mixed well by inverting the tube several times. The mixture was then incubated at 37 °C overnight. About 100 µL of 4.5 M NaCl was added and mixed well. Then, about 225 µL chloroform was added followed by shaking for 10 min by hand. The mixture was centrifuged at 14,000 rpm for 10 min. About 200 µL of the aqueous phase was transferred into a new tube and an equal volume of iso-propanol was added followed by mixing by inverting the tube several times. The mixture was centrifuged at 14000 rpm for 15 min and the supernatant was discarded. About 500 µL of 70% ice-cold ethanol was added to the tube and kept at room temperature for 15 min. The tube was then centrifuged at 14000 rpm for 15 min. The supernatant was discarded, and the DNA pellet was dried at room temperature for about 10 min and re-suspended in 50 µL TE buffer. The tube was incubated overnight at 37 °C. Then, the DNA was homogenized by pipetting 30×. The purity of extracted DNA was measured by spectrophotometer (NanoDrop 2000c, ThermoFisher Scientific) and an OD ratio at A260/A280 of ~1.8 was considered as good. Good quality DNA was stored at −20 °C until further analysis. 

### 2.4. Primers and Restriction Enzymes 

Two sets of primers with different restriction enzymes were used for *Mx* gene detection as reported by [31,32]. Primer sequences used for *Mx* gene detection, restriction enzymes, and restriction patterns of DNA are included in Table 2. Primers used for AIV detection, H, and N subtyping as well as detection of NDV were reported earlier [33,34,35,36,37]. 

### 2.5. Amplification of Mx Gene by Polymerase Chain Reaction (PCR) 

The 25 µL PCR reaction mixture consisted of 12.5 µL of 2× master mix (GENE Amp Fast PCR Master mix (2×)), 2 µL sample (~50 ng/µL), 0.2 µL Taq DNA polymerase, 0.5 µL forward primer, 0.5 µL reverse primer (each 20 pmole), and 9.3 µL DNAse free water. Thermal conditions for primers were reported by Sironi et al. [31] and included initial denaturation for 5 min at 95 °C, followed by 35 cycles of denaturation at 94 °C for 1 min, annealing at 60 °C for 1 min, extension at 72 °C for 1 min, and a final extension at 72 °C for 10 min. Gene Atlas (Model: G02, Japan) was used for thermal cycling. Amplicons were analyzed by electrophoresis in 1.5% agarose gel stained with ethidium bromide. A molecular weight marker was used as standard. 

### 2.6. Mx Gene Diversity Analysis by Restriction Fragment Length Polymorphism (RFLP) Analysis 

The PCR products obtained using primers reported by Sironi et al. [31] were digested with restriction enzyme *Hpy*8I (NEB, Hertfordshire, United Kingdom). PCR products obtained using NE-F2 and NE-R2/R primers reported by Seyama et al. [32] were digested with the *Rsa*I (Biolabs, MA, USA) and those obtained using NE-F2 and NER2/S primers were digested with *Ssp*I (BioLabs, MA, USA). The digestion reaction (10 μL) consisted of nuclease free water (3 μL), compatible 10× buffer (1 μL), specific restriction enzyme (1 μL), and the PCR product (5 μL). The reaction mixture was incubated at 37 °C in a water bath for one hour. Upon digestion, the products were electrophoresed in 3% agarose gel containing ethidium bromide. DNA was visualized with a transilluminator. Diversity analysis of *Mx* gene and genotyping of chickens based on the *Mx* gene were made based on restriction patterns, as shown in Table 2.

### 2.7. Anti-AIV Antibody Detection 

Serum samples (*n* = 512) from chicken blood were subjected to spinning at 1500 rpm. Clarified sera were stored at −20 °C until testing. The sera were tested for anti-AIV antibody by commercially available indirect ELISA kit (IDEXX AI MultiS-Screen Ab Test, ME, USA) in accordance with the manufacturer’s instructions. 

### 2.8. Virus Isolation and Identification 

Cloacal swab samples were vortexed and centrifuged at 15,000 rpm for 3 min. Clear supernatants were collected and stored at −20 °C until use. Virus isolation was performed in 9–12-day-old embryonated chicken eggs (ECE). About 200 µL of the sample was inoculated into each egg through the allantoic cavity. For each sample, two ECE were used. Upon inoculation, the eggs were incubated at 37 °C for 4 days and checked twice daily. Death within 24 h of inoculation was considered as accidental and discarded. Upon incubation, the eggs were chilled and the allantoic fluid was collected. The fluid was screened by hemagglutination (HA) test using 0.5% chicken red blood cell (CRBC) to determine the presence of HA viruses such as AIV and NDV [38,39]. HA negative samples were passaged at least once more in ECE before considering the sample as negative for virus isolation. For virus identification, RNA was extracted from the HA-positive allantoic fluid followed by reverse transcription polymerase chain reaction (RT-PCR) using virus-specific primers. Two viruses, AIV and NDV, were considered for identification and differentiation by RT-PCR [33,34,35,36,37]. 

### 2.9. Statistical Designs/Analytical Methods Used 

Allele frequencies, genotype frequencies, and Hardy–Weinberg equilibria were calculated in accordance with the method reported in Nei and Kumar [40]. The General Linear Model (GLM) procedure of SAS 9.1.0 software (SAS Institute Inc., Cary, NC, USA) was used for association analysis between different *Mx* gene genotypes and avian influenza virus infection. 

## 3. Results

### 3.1. General Properties of Sampled Chickens

Samples for indigenous chickens were collected from 3–5 villages of each upazila, commercial layer chicken samples were collected from eight farms, and two batches of Sonali chickens were collected from the same hatchery. The age of the sampled chickens ranged from 1 to 16 months. The chickens were apparently healthy during sample collection (Table 1). Un-sexed day-old Sonali chicks were purchased from the hatchery and reared at NIB until one month of age then blood and cloacal swab samples were collected. History of AIV vaccination in commercial layer chickens as well as parents of Sonali chicken was recorded during sample collection. Sampling was mostly conducted in cooler months of the year considering the higher possibility of getting AIV. 

### 3.2. Detection of Mx Gene 

A total of 512 DNA samples isolated from the cellular portion of the blood of indigenous and commercial chickens were subjected to PCR to amplify a ~299 bp fragment and the ~100 bp products of the *Mx* gene. Samples were amplified with two sets of primers and results were consistent throughout.

### 3.3. Diversity of Mx Gene and Genotyping of Chickens

PCR products, ~299 bp fragment of the *Mx* gene, were digested using the *Hpy*8I restriction enzyme and the diversity of the *Mx* gene was determined based on digestion patterns. Three genotypes (the homozygous resistant AA, the homozygous sensitive GG, and the heterozygous AG) and two alleles (A and G) of the *Mx* gene were detected in the tested chicken samples (Table 3). At the individual chicken type, the genotype frequency was between 0.03 and 1.0, whereas the allele frequency was between 0 and 1.0. The resistant AA genotype (0.48) was more dominant than the heterozygous AG (0.33) and the sensitive GG (0.19) genotypes. Moreover, the A allele (0.64) was more frequent than the G allele (0.36) in the studied populations.

In indigenous chickens, the overall genotype frequency was 0.45, 0.45, and 0.10 for the AA, the AG, and the GG genotypes, respectively. Moreover, the allele frequency was 0.68 and 0.32 for the A and G alleles, respectively. The resistant AA genotype was more frequent in Common deshi (0.89), followed by Naked neck (0.49), Aseel (0.22), and Hilly (0.11) chickens. Aseel was found to carry a higher frequency of the heterozygous AG genotype (0.71) than Hilly (0.65), Naked neck (0.44), and Common deshi (0.11). The frequency of the sensitive GG genotype was higher in Hilly (0.24) than in Common deshi (0.00) chickens. The frequency of the A allele was higher than the G allele in the indigenous group of chickens with the exception of the Hilly chicken. Higher heterozygosity than expected was observed in all indigenous chickens indicating a possible isolate breaking effect or a cross breeding history. Only Aseel and Hilly chickens were found to follow Hardy–Weinberg equilibrium (*p* < 0.05) in the studied population. In the commercial layer group of chickens, the overall genotype frequencies were 0.54, 0.01, and 0.45 for the AA, the AG, and the GG genotypes, respectively. The frequency of alleles A and G were 0.54 and 0.45, respectively. The breed of commercial layer chickens sampled from Savar, Dhaka region (Breed denoted as X) was found to carry a 100% resistant (AA) genotype. The breed from the Gazipur Sadar region (Breed denoted as Y) was found to carry a 100% sensitive (GG) genotype of the *Mx* gene. The results from commercial layer chicken samples were further confirmed by PCR-RFLP with mismatch primers as shown in Table 2. In the case of the Sonali chicken, the AA genotype (0.57) and the A allele (0.59) were more frequent and significant differences (*p* < 0.05) were found between the observed (0.03) heterozygosity and the expected (0.48) heterozygosity. 

### 3.4. Serological Status of Chickens

**Figure 2:**
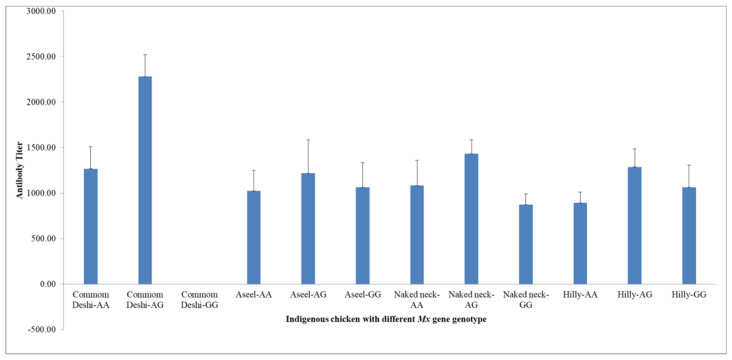
Anti-AIV antibody titers detected in different *Mx* gene genotype types of various indigenous chickens. The titer was detected by commercially available ELISA kit (IDEXX AI MultiS-Screen Ab Test, ME, USA).

A total of 512 serum samples were tested for anti-AIV antibodies by indirect ELISA. The average optical density was used to calculate the antibody titer. Anti-AIV antibodies were detected in 230/512 (44.92%) of the samples (Table 4). The prevalence ranged between 26% and 87.5% in both indigenous and commercial chickens. The anti-AIV antibody was detected in all four groups of non-vaccinated indigenous chickens and prevalence ranged between 27.37% and 67.65%, with an overall prevalence of 37.90%. Moreover, 27.37%, 30.48%, 35.78, and 67.65% of the samples of Hilly, Common deshi, Naked neck, and Aseel, respectively, were positive for the anti-AIV antibody. In commercial layers and Sonali chickens, the prevalence ranged between 26% to 87.5%. Only commercial layer chickens and parents of Sonali chicken had a history of AIV vaccination, which was recorded at the time of sample collection. The antibody titers varied within and among different genotypes of chickens. Lower antibody levels were detected in the homozygous resistant (AA) and sensitive (GG) alleles than in the heterozygous (AG) alleles (Figure 2). Among all indigenous chickens, higher antibody titers were found in *Mx* AG genotype chickens. No specific trends were found in the AA and the GG genotypes. 

### 3.5. Virological Status of Chickens

For virus isolation, processed swab samples were cultured in 9–12-day chicken embryos. Viral presence in the allantoic fluid was detected by the HA test. Identification of the virus was performed by RT-PCR using AIV-specific and NDV-specific primers. A total of 17 hemagglutinating viruses were isolated from 512 samples with a 3.29% (17/512) isolation rate. However, AIV was isolated and identified from only 0.39% (2/512) samples and NDV from 2.93% (15/512) samples (Table 5). Two AIVs were isolated from samples of Common deshi and Naked neck chicken samples collected from Savar, Dhaka and Madhupur, Tangail, respectively. H and N subtyping of two AIVs by RT-PCR revealed that both were H1N1.

### 3.6. Association Analysis

Data related to the *Mx* gene genotype and the anti-AIV antibody detected in corresponding chicken sera were used for analysis of a possible association between the different *Mx* allelic genes and AIV infection in poultry. Antibody detection in chickens with a history of no vaccination was considered the result of natural AIV infection. The general linear model (GLM) procedure of SAS 9.1.0 software (SAS Institute Inc., Cary, NC, USA) was used for association analysis. All three genotypes of chickens were found to be seropositive. Overall, 40.16, 39.77, and 65.98% chickens of the AA (resistant), the AG (heterozygous), and the GG (sensitive) genotypes, respectively, had anti-AIV antibodies (Table 4). All genotypes were found to be significantly (*p* < 0.001) associated with the anti-AIV antibodies and thus with AIV infection (Table 6). Moreover, the H1N1 virus was isolated from Common deshi chicken carrying the resistant AA genotype. It is noteworthy that the anti-AIV antibody (very low titer) was also detected in the serum of the Common deshi chicken. The H1N1 virus isolated from Naked neck chickens was of the heterozygous AG genotype and no anti-AIV antibody was detected. These results indicate that even the resistant genotype (AA) of the *Mx* gene was unable to resist the infection.

## 4. Discussion 

Chickens are natural hosts for the influenza virus and many other viruses. Viral infections can cause serious illness or death in chickens. The AIV is also infectious to humans and has led to acute conditions in some cases [41]. Selective breeding of AIV-resistant chickens would be beneficial for both the livestock industry and human health. Therefore, the present study was conducted to measure the AIV resistance *Mx* gene and its diversity in chickens. The relationship of the *Mx* gene with AIV infection has also been determined. For this purpose, chicken DNA, serum samples, and cloacal swab samples collected from different types of chickens from various regions of the country were analyzed by various techniques. We have amplified specific-size DNA and genotyped the *Mx* gene from all samples using the PCR-RFLP method reported earlier [31]. Sironi et al. [31] examined 127 samples of commercial broiler, White Leghorn, and New Hampshire chicken and genotyped all samples and then confirmed the results by *Mx* gene sequencing. Our findings are consistent with the findings of Sironi et al. [31]. We also confirmed our results with two other sets of primers and restriction enzymes reported previously by Seyama et al. [32], who developed a PCR-RFLP technique to determine *Mx* gene, its variation, and to genotype all samples from 36 strains of 17 chicken breeds. Thus, the PCR RFLP technique was suitable for genotyping of chickens based on *Mx* gene diversity analysis. 

An outbreak of HPAI first occurred in Bangladesh in 2007, and since then outbreaks have continued to occur across the country and were mostly reported from commercial chickens (*n* = 499) rather than indigenous chickens (*n* = 57) [9]. Some host factors might have been responsible for the lower occurrences of HPAI in indigenous poultry in Bangladesh. The presence of the *Mx* gene in chickens may be one of these antiviral host factors that allow indigenous chickens to resist the virus. The *Mx* gene coding protein was shown to be induced by interferon (IFN) and to inhibit the replication of RNA viruses [18,42]. Watanabe, [43] studied chicken Mx cDNAs from different breeds to see whether these chickens carried resistant *Mx* genes to the vesicular stomatitis virus (VSV) infection, and a one amino acid substitution in position 631 was responsible for differences in the antiviral activities of chicken Mx proteins. Asparagine (Asn) at 631 corresponded to positive antiviral activity, whereas serine (ser) corresponded to negative antiviral activity [18,43,44]. However, Benfield et al. [20] reported that the breed, Shamo, which has an asparagine in position 631 of the Mx protein is void of activity against H5N1. Similarly, Bernasconi et al. [45] reported that the chicken Mx protein in German White Leghorn lacked antiviral activity for both influenza and VSV. In the present study, we determined the variation/diversity of the *Mx* gene and found that three types of *Mx* gene, namely, resistant (AA), sensitive (GG), and heterozygous (AG), existed in the tested chicken samples. Diversity in *Mx* gene was found not only within the groups but also among the groups (Table 3). Our findings are consistent with the findings of Seyama et al. [32]. They investigated 271 DNAs of commercial and indigenous chickens from different sources and found resistant, sensitive, and heterozygous *Mx* gene alleles within and between groups of chickens. Similar results were also reported by other investigators [19,46]. They also reported that indigenous chickens have a higher frequency of the resistant-type *Mx* gene allele. The findings in the present study regarding distribution of *Mx* gene were consistent with the findings of Seyama et al. [32]. In our study, 48%, 19%, and 33% of the tested samples represented resistant, sensitive, and heterozygous *Mx* gene alleles, respectively. Seyama et al. [32] reported 33.95% resistant, 52.40% sensitive, and 13.65% heterozygous alleles in the tested samples (*n* = 271). The present findings are also consistent with previous findings in which 42.86% homozygous resistant, 42.86% homozygous sensitive, and 14.29% heterozygous *Mx* gene alleles were reported [27]. 

Serological surveys are among the most direct and informative technique available to infer the dynamics of a population’s susceptibility and its levels of immunity. We have conducted anti-AIV antibody surveys in 512 chicken serum samples. Only commercial layer chickens and parents of Sonali chicken samples had a history of vaccination. It is worth mentioning that no routine vaccination programs are conducted against avian influenza in indigenous chicken production systems in Bangladesh. The detection of anti-AIV antibodies in these birds indicated that AIVs circulate in the chicken flocks. Moreover, the antibodies detected could only have been derived from seroconversion following natural infection with the viruses. Consequently, the infected birds might be playing a crucial role in the epidemiology of the disease through shedding of the viruses into the environment. The overall presence of AIV-specific antibodies was 44.92%. However, seroprevalence in non-vaccinated indigenous chickens was 37.9% (Table 4). Such findings are in agreement with serological survey results that were reported by Jahangir et al. [47] from Cox’s Bazar (38.6%), although they recorded lower seroprevalence in other districts of Bangladesh (Barisal, 32.3% and Bogra, 14%). Lower seroprevalence in indigenous chickens was also reported by Rahman et al. [48] (14.4%), Nooruddin et al. [49] (8.92%), and Biswas et al. [50] (20%). Higher antibody titers were detected in samples derived from chickens with a vaccination history. Differences in the management system and agroecological conditions of the study sites might be responsible for this variation. The indigenous chickens in the villages studied were allowed to scavenge with ducks in the yard and in the crop fields, where there were numerous and different species of wild birds. This husbandry practice might have contributed to the natural infection of the indigenous chickens and might explain the high prevalence detected. These findings suggest that AIV is circulating among backyard chickens in the studied populations. The serological studies conducted before the first outbreak of HPAI provided evidence that smallholders’ chickens in Bangladesh might have been harboring some subtypes of influenza A viruses in or before 2003 [47,50]. 

We have investigated cloacal swab samples collected from indigenous chickens as well as commercial layer and Sonali chickens for the presence of hemagglutinating viruses AIV and NDV. For the gold standard technique, we have used chicken embryos to culture viruses in cloacal swab samples followed by the detection of hemagglutinating viruses by HA test. Later, the viruses were identified by RT-PCR using AIV-specific as well as NDV-specific primers. The hemagglutinating virus isolation rate was 3.29%. However, AIV isolation rate was only 0.39%. Additionally, 2.93% of samples were positive for NDV (Table 5). Since the first outbreak, HPAI has continued to occur throughout the country [9]. Gerloff et al. [6] performed influenza virus surveillance, poultry outbreak investigations, and genomic sequencing to understand the ecology and evolution of low pathogenic avian influenza (LPAI) A viruses in Bangladesh from 2007 to 2013. They analyzed 506 avian specimens collected from poultry in live bird markets and backyard flocks and isolated fifty AIV with an overall isolation rate of 9.88%. The most frequently identified LPAI subtypes were H9N2, H11N3, H4N6, and H1N1. The less frequently detected subtypes included H1N3, H2N4, H3N2, H3N6, H3N8, H4N2, H5N2, H6N1, H6N7, and H7N9. In our study, we isolated only one subtype of AIV (the H1N1). Khan et al. [51] also conducted a longitudinal AIV surveillance in domestic waterfowl and market environments during 2007–2012 and reported a 4.4% (191/4308) prevalence of AIV in waterfowl. The subtypes of AIVs detected in waterfowl and environmental samples included H1N1, H1N3, H3N2, H3N6, H3N8, H4N1, H4N2, H4N6, H5N1, H5N2, H6N1, H7N9, H9N2, H11N2, H11N3, and H11N6. Future studies could utilize challenge experiments to directly assess the association of AIV infection and the *Mx* gene allele.

The role of the host’s genetic background in mounting immune responses to in vivo infections of chicken with AIV was investigated by Sironi et al. (2011). They examined the *Mx* gene allele of chickens and responses to the HPAI A/Chicken/Italy/13474/99 H7N1 virus. They could not find a significant association between the *Mx* gene genotype and the response of the birds to AIV. However, they could not rule out the possible influence of other polymorphisms within the *Mx* gene in the in vivo antiviral activity. In a recent study Lee et al. [52] showed that *MX1*, *STAT1*, *IRF7*, and *TLR3* genes were upregulated in resistant Ri chicken lines in comparison to susceptible lines. The immune response to infection in HPAIV-resistant Ri chicken lines was different than in HPAIV-susceptible Ri chicken lines. This finding clearly indicates that multiple genes, and not just the Mx gene, are involved in influenza resistance in Vietnamese chickens as they likely do in chickens native to Bangladesh. Association analysis between the *Mx* gene allele and anti-AIV antibody prevalence in chickens in our study revealed that the *Mx* gene allele had no apparent effect on immune response. Detection of antibodies to AIV in non-vaccinated birds indicated exposure to AIV with a resultant seroconversion. Though disease-resistant chickens with the *Mx* gene have been proposed [53], we could not find *Mx* gene genotype-specific effect. The lower antibody levels in the homozygous resistant (AA) versus the heterozygous (AG) allele might be due to the lower reactivity of resistant-allele chickens to viruses than the sensitive-allele chickens. Similar results were reported even after vaccination [54], in which the authors reported that chickens with homozygous resistant alleles (AA) exhibited weaker antibody responses to the H5N2 AIV vaccine than those of the heterozygote (AG) alleles. The lowest titers were also reported in the resistant AA allele chickens even after booster vaccinations, indicating negative effects of the resistant allele A on the response of the chicken to AIV [55,56,57].

## Figures and Tables

**Table 1:** Distribution and characteristics of sampled chickens (*n* = 512).

Sampling Location	Chicken Type	No. of Samples *	No. of Villages/ Farms	Age (mo.)	Sex	Health Condition
Indigenous Chickens						
Savar, Dhaka	Common deshi	105	3	6–11	M = 40 F = 65	Apparently healthy
Brahmanbaria Sadar	Aseel	68	4	9–14	M = 30 F = 38	Apparently healthy
Madhupur, Tangail	Naked neck	109	5	2–6	M = 29 F = 80	Apparently healthy
Bandarban Sadar	Hilly	95	4	6–12	M = 40 F = 55	Apparently healthy
Commercial Chickens						
Savar, Dhaka	Commercial (Breed X)	50	5	12–16	F = 50	Apparently healthy
Gazipur Sadar	Commercial (Breed Y)	45	3	11–16	F = 45	Apparently healthy
Savar, Dhaka	Sonali	40	2	1	M = 14 F = 26	Apparently healthy
Total		512				

* Blood and cloacal swab samples were collected from chickens. Serum and blood cells were separated from each blood sample. *Mx* gene and its diversity were determined using DNA extracted from blood cells, whereas serum was tested for anti-AIV antibody detection by indirect ELISA. Cloacal swab samples were tested for virus isolation and identification.

**Table 2:** Primers and restriction enzymes for *Mx* gene amplification and diversity analysis.

Primer Sequence 5′-3′	PCR Product Size (bp)	Restriction Enzyme	Fragment Length upon Digestion	Reference
F: GCACTGTCACCTCTTAATAGA	299	*Hpy*8I	AA: 299 AG:299, 200, 99 GG: 200, 99	[31]
R: GTATTGGTAGGCTTTGTTGA				
NE-F2: CCTTCAGCCTGTTTTTCTCCTTTTAGGAA	100	*Rsa*I	AA: 100 AG:100, 73, 27 GG: 73, 27	[32]
NE-R2/R: CAGAGGAATCTGATTGCTCAGGCGTGTA				
NE-F2: CCTTCAGCCTGTTTTTCTCCTTTTAGGAA	100	*Ssp*I	AA: 73, 27 AG: 100, 73, 27 GG: 100	
NE-R2/S: CAGAGGAATCTGATTGCTCAGGCGAATA				

**Table 3:** *Mx* gene diversity in indigenous and commercial chickens.

Chicken Type	Total Samples	Genotype Frequency (No.)			Allele Frequency		Ho	He	HWE
		AA	AG	GG	A	G			
Indigenous Chickens									
Common Deshi	105	0.89 (93)	0.11 (12)	0.00 (0)	0.95	0.05	0.11	0.10	*p* > 0.05
Aseel	68	0.22 (15)	0.71 (48)	0.07 (5)	0.58	0.42	0.71	0.49	*p* < 0.05
Naked Neck	109	0.49 (53)	0.44 (48)	0.07 (8)	0.71	0.29	0.44	0.41	*p* > 0.05
Hilly	95	0.11 (10)	0.65 (62)	0.24 (23)	0.44	0.56	0.65	0.49	*p* < 0.05
Subtotal Indigenous Chicken	377	0.45 (171)	0.45 (170)	0.10 (36)	0.68	0.32	0.45	0.43	
Commercial Chickens									
Commercial Chicken- X	50	1.0 (50)	0 (0)	0 (0)	1.0	0	0	-	-
Commercial Chicken-Y	45	0 (0)	0 (0)	1.0 (45)	0	1.0	0	-	-
Sonali	40	0.57 (23)	0.03 (1)	0.40 (16)	0.59	0.41	0.03	0.48	*p* < 0.05
Subtotal Commercial	135	0.54 (73)	0.01 (1)	0.45 (61)	0.54	0.45	0.01	0.48	

Ho: Observed heterozygosity, He: Expected heterozygosity, HWE: Hardy–Weinberg equilibrium, -: Not performed.

**Table 4:** Anti-AIV antibody prevalence in different *Mx* gene genotypes of chickens (*n* = 512).

Chicken Type	% Ab Positive	No. of AA Genotype	% Ab Positive	No. of AG Genotype	% Ab Positive	No. of GG Genotype	% Ab Positive
Non-vaccinated chickens							
Common Deshi	30.48 (32/105) *	93	30.11 (28/93)	12	33.33 (4/12)	0	0
Aseel	67.65 (46/68)	15	80.00 (12/15)	48	60.42 (29/48)	5	100 (5/5)
Naked Neck	35.78 (39/109)	53	37.74 (20/53)	48	35.42 (17/48)	8	25.00 (2/8)
Hilly	27.37 (26/95)	10	40.00 (4/10)	62	27.42 (17/62)	23	21.74 (5/23)
Subtotal	37.90 (143/377)	171	37.43 (64/171)	170	39.41 (67/170)	36	33.33 (12/36)
Vaccinated Chickens							
Commercial X	26.0 (13/50)	50	26.00 (13/50)	0	0	0	0
Commercial Y	86.67 (39/45)	0	0	0	0	45	86.67 (39/45)
Sonali	87.50 (35/40)	23	91.30 (21/23)	1	100 (1/1)	16	81.25 (13/16)
Sub-total	64.44 (87/135)	73	46.58 (34/73)	1	100 (1/1)	61	85.25 (52/61)
Grand Total	44.92 (230/512)	244	40.16 (98/244)	171	39.77 (68/171)	97	65.98 (64/97)

* Number of samples positive for anti-AIV antibody/Number of tested samples.

**Table 5:** Isolation and identification of hemagglutinating viruses from cloacal swab samples of indigenous and commercial layer chickens (*n* = 512).

Chicken Type	Tested Samples	HA-Positive Samples	Samples with Virus		AIV Subtype/*Mx Gene* Genotype
			AIV	NDV	
Common Deshi	105	5	1	4	H1N1/AA
Aseel	68	4	0	4	
Naked Neck	109	5	1	4	H1N1/AG
Hilly	95	3	0	3	
CommercialX	50	0	0	0	
CommercialY	45	0	0	0	
Sonali	40	0	0	0	
Total (%)	512	17 (3.29)	2 (0.39)	15 (2.93)	

**Table 6:** Least squares mean (LSM) and standard errors (SE) for *Mx* gene genotype and anti-AIV antibody prevalence (AIV infection in non-vaccinated birds) in chickens.

Locus	Genotype	Anti-AIV Antibody Prevalence
g.2032 A > G	AA (243)	1.399 ± 0.031 ^A^
	AG (171)	1.397 ± 0.037 ^AB^
	GG (97)	1.659 ± 0.049 ^AB^
		*p* < 0.01

Different superscripts of capital letters in the same column indicate significant difference at *p* < 0.01 between genotypes and anti-AIV antibody prevalence.

## Data Availability

All relevant data is included in the article.
